# Brain and cortisol responses to smoking cues are linked in tobacco-smoking individuals

**DOI:** 10.1111/adb.13338

**Published:** 2023-12

**Authors:** Timothy J. Wanger, Fernando B. de Moura, Rebecca Ashare, James Loughead, Scott Lukas, Caryn Lerman, Amy C. Janes

**Affiliations:** 1 McLean Imaging Center, McLean Hospital, Belmont, Massachusetts, USA; 2 Harvard Medical School, Boston, Massachusetts, USA; 3 Department of Psychiatry, Perelman School of Medicine, University of Pennsylvania, Philadelphia, Pennsylvania, USA; 4 Department of Psychology, University at Buffalo, Buffalo, New York, USA; 5 Department of Psychiatry, University of Pennsylvania, Philadelphia, Pennsylvania, USA; 6 Norris Comprehensive Cancer Center, University of Southern California, Los Angeles, California, USA; 7 Neuroimaging Research Branch, National Institute on Drug Abuse (NIDA), Intramural Research Program, National Institutes of Health, Baltimore, Maryland, USA

**Keywords:** cortisol, cue reactivity, insula

## Abstract

Cues associated with smoking can induce relapse, which is likely driven by cue-induced neurobiological and physiological mechanisms. For instance, greater relapse vulnerability is associated with increases in cue-induced insula activation and heightened cortisol concentrations. Determining if there is a link between such cue-induced responses is critical given the need for biomarkers that can be easily measured in clinical settings and used to drive targeted treatment. Further, comprehensively characterising biological reactions to cues promises to aid in the development of therapies that address this specific relapse risk factor. To determine whether brain and cortisol responses to smoking cues are linked, this study recruited 27 nicotine-dependent tobacco-smoking individuals and acquired whole-brain functional activation during a cue reactivity task; salivary cortisol was measured before and after scanning. The results showed that increases in blood-oxygen-level-dependent activation in the right anterior insula and right dorsolateral prefrontal cortex (DLPFC) when viewing smoking versus neutral cues were positively correlated with a post-scan rise in salivary cortisol concentrations. These brain regions have been previously implicated in substance use disorders for their role in salience, interoception and executive processes. These findings show that those who have a rise in cortisol following smoking cue exposure also have a related rise in cue-induced brain reactivity, in brain regions previously linked with heightened relapse vulnerability. This is clinically relevant as measuring cue-induced cortisol responses is a more accessible proxy for assessing the engagement of cue-induced neurobiological processes associated with the maintenance of nicotine dependence.

## 1 | INTRODUCTION

Tobacco use remains a significant public health concern given that available interventions are limited in their capacity to help prevent individuals from returning to use.^1^ Compelling evidence indicates that conditioned smoking cues are a catalyst for relapse even in those motivated to quit.^2–6^ Therefore, developing a more comprehensive understanding of factors associated with enhanced cue reactivity remains of paramount importance. A great deal of cue reactivity research has used functional magnetic resonance imaging (fMRI), and a meta-analysis of those results revealed that drug cues induce activation in a range of regions associated with cognition, attention and salience.^7^ Across multiple drug classes, relapse vulnerability is associated with cue-induced activation in the cingulate cortex, dorsal striatum, prefrontal cortices and the insula.^8^ Of these regions, mounting evidence shows that the insula is consistently activated by smoking-related cues and enhanced activity in this region has been linked to a greater likelihood of relapse.^4,5,9,10^ In addition to activating specific brain regions, drug cues elicit increases in salivary cortisol concentrations, which has been independently linked to poor cessation outcome.^11–13^ Whether these two independent measures of cue reactivity are linked is unclear and the present study was conducted to provide additional insight into this brain–cortisol relationship during tobacco smoking-related cue reactivity.

Glucocorticoids, such as cortisol (or corticosterone in rodents), play a role in the body’s response to stressful events,^14,15^ but also are involved in the regulation of numerous immunological, homeostatic and cognitive/behavioural processes.^16^ In translational models of substance use disorders, plasma glucocorticoid concentrations are elevated following drug^17–19^ and drug cue exposure.^12,20^ Glucocorticoids also have an established role in mediating drug use as inactivation of glucocorticoid receptors eliminate drug self-administration in mice^21^ and glucocorticoid receptor antagonists prevent both excessive drug self-administration and craving in humans.^22^ In humans, acute intravenous administration of cortisol triggers a drug craving response in cocaine-dependent individuals that is similar to the craving response following a priming dose of cocaine.^23^ Therefore, glucocorticoids’ broad role in the body’s physiological response to environmental stimuli may directly or indirectly mediate drug-seeking behaviour, as is seen in the association between increased drug cue-induced cortisol and drug use.^13^

Despite strong evidence from rodent and human studies indicating that cue-induced brain activation and cortisol responses maintain drug-seeking behaviours, a direct relationship between these two types of cue-related responses has yet to be established. Identifying a connection between the brain reactivity to smoking cues and cortisol responsivity may represent a critical piece of information when determining which individuals are predisposed to react strongly to smoking cues and therefore will struggle with quitting and subsequently may return to use. Given salivary cortisol is easier to measure than brain reactivity, such links may also provide correlates of brain function that are more clinically accessible for guiding person-centered therapy. As such, this study aimed to directly establish a relationship between smoking-cue-induced changes in salivary cortisol and specific regional brain activation patterns. The results from this study will help to clarify whether brain and endocrine responses to drug cues covary in people who regularly smoke tobacco.

## 2 | METHODS

### 2.1 | Overall protocol

Participants reported to the laboratory for a single study visit that included blood-oxygen-level-dependent (BOLD) imaging during exposure to both smoking-related and neutral cues. Salivary cortisol concentrations were quantified at baseline (when participants first arrived for the visit) and again before and after the imaging session. The neuroimaging experiment used a within-subjects design, contrasting the smoking cue and neutral cue conditions. Preprocessing and analysis of fMRI data were conducted using tools from the functional magnetic resonance imaging of the brain (FMRIB) Software Library (FSL 10; www.fmrib.ox.ac.uk/fsl). Using FSL, the change in cortisol from pre- to post-scan for individuals was regressed against the whole-brain voxel-wise contrast of smoking and neutral cues.

## 3 | PARTICIPANTS

The final sample included 27 participants who were (on average) moderately nicotine-dependent. Nicotine dependence was estimated using the Fagerström Test for Nicotine Dependence (FTND^24,25^), where scores between 4 and 6 indicate moderate dependence (for a summary of demographic and smoking information, see Table 1).

Participants were included if they reported smoking at least five cigarettes daily for at least the past 6 months. At the time of screening, current smoking status was confirmed biochemically by measuring expired air carbon monoxide (CO) ≥ 6 ppm (Bedfont Micro IV Smokerlyzer, Bedfont Scientific, Kent, England), and absence of amphetamines, barbiturates, benzodiazepine, cocaine, marijuana, methadone, methamphetamine, methylenedioxymethamphetamine (MDMA), opiates, oxycodone and phencyclidine (PCP), was confirmed with urine samples, whereas lack of recent alcohol use was confirmed with breath samples (Quick-Tox11 Panel Drug Test Card; Branan Medical Corporation, Irvine, CA; and Alco-Sensor IV; Intoximeters, St. Louis, MO, USA). Participants were excluded if they had been diagnosed with a substance use disorder other than nicotine in the prior 6 months, current major depressive disorder, lifetime diagnosis of bipolar disorder or schizophrenia or positive pregnancy test. Participants were instructed to maintain their regular smoking patterns prior to the study visit and smoked one of their own cigarettes 1.5 h prior to the fMRI scan. Neuroimaging data for anatomical, resting state and functional activation in response to smoking and neutral cues were obtained for each subject in that order. Only data from the cue reactivity paradigm is reported here.

### 3.1 | Cortisol

Approximately 1.5–2.0 mL of saliva was collected at three time points using a cotton swab that participants were instructed to place between their tongue and cheek for approximately 2–3 min until the swab was completely saturated. The saliva swab was then placed in a plastic tube and stored at −20°C. Saliva samples were assayed using salivary high-sensitivity cortisol enzyme immunoassay (EIA) kits (Salimetrics LLC, State College, PA, USA). Salivary cortisol was collected when participants first arrived for the visit. Additional cortisol assessments were then acquired, approximately 15 min before entering the scanner, and 15 min after scanning. Due to prior evidence that human cortisol levels vary across the day,^26^ linear regression was used to test whether baseline cortisol or change in cortisol levels were associated with the time of the scan.

### 3.2 | Cue reactivity task

A cue reactivity task as described in prior work^27–29^ was used in the present study. Briefly, there were five event-related runs during which participants viewed 10 smoking, 10 non-smoking and 2 target images in a pseudorandom order (no two images of the same type appeared in succession). Each image was presented for 4 s, with a jittered intertrial interval of 6–14 s, mean = 10 s. Smoking images were naturalistic scenes depicting people smoking, people holding cigarettes and smoking paraphernalia. Non-smoking images were matched in content with the smoking images but lacked the smoking cue element. Target images included animals and were included to verify attention via a button press. Craving was estimated before and after the cue reactivity task using the Questionnaire of Smoking Urges (QSU-brief^30^).

### 3.3 | Data acquisition

Neuroimaging data were acquired on a Siemens Magnetom 3 T Prisma with a 64-channel head coil. Multi-echo magnetisation-prepared rapid acquisition gradient echo (MPRAGE) structural images were acquired with the following parameters: TR = 2.53 s, TE = 3.3 ms, slices = 128, matrix = 256 × 256, flip angle = 7°, resolution = 1.0 mm × 1.0 mm × 1.33 mm. Gradient echo-planar images were acquired during cue reactivity. Cue reactivity acquisition used the following parameters: TR = 0.72 s, TE = 30 ms, flip angle = 66°, slices = 66, voxel size = 2.5 mm isotropic, GRAPPA acceleration factor = 2, multi-band acceleration factor = 6.

### 3.4 | fMRI preprocessing

Using FSL, the first five volumes of each run were removed to allow for signal stabilisation. Functional data pre-processing included the following: motion correction with MCFLIRT, brain extraction using BET, slice timing correction, spatial smoothing with a Gaussian kernel of full-width half-maximum 6 mm, and high-pass temporal filter with Gaussian-weighted least-squares straight-line fitting with σ = 100 s. Consistent with our prior work^28^ artefacts due to motion and intensity spiking were identified and removed prior to pre-processing in FSL (https://github.com/bbfrederick/spikefix). Subject specific data were registered to the MNI152 2 mm standard space template (Montreal Neurological Institute, Montreal, QC, Canada), and fMRI data were transformed into standard space at 2 mm isotropic resolution using registration transformation matrices (FNIRT).

### 3.5 | Cue reactivity analysis

Using FSL, a first-level GLM including three regressors corresponding to smoking, neutral and target image presentation was conducted for each of the participant’s five cue reactivity runs. A baseline condition was collected, whereas participants viewed a white fixation cross on a black background but was not specifically modelled. Task regressors were convolved with the standard gamma hemodynamic response function. Confound regressors also were included to model motion effects (*x*, *y* and *z* translation and rotation motion). Another regressor was included that represented motion/intensity artefacts identified and removed prior to pre-processing (https://github.com/bbfrederick/spikefix). Using this technique, volumes were removed if there was a 3% signal change across 150 voxels. Overall, motion in this sample was low as the average absolute mean displacement was 0.152 mm +/− 0.164. No participants were excluded based on motion. First-level results were combined (across the five blocks) using a second-level fixed effects analysis to generate average brain reactivity for each individual participant.

### 3.6 | Group-level cue reactivity and cortisol analysis

Changes in cortisol concentration were calculated by subtracting the post-scan value (immediately after scanning) from the pre-scan score (15 min before scanning). These values were demeaned and entered into a group level analysis using a mixed effects model (FLAME 1). Changes in cortisol levels were correlated with whole brain activity for the smoking cue > neutral cue contrast. The average smoking cue > neutral cue brain reactivity was also assessed without evaluating the impact of the change in cortisol. In FSL, clusters were identified^31^ by using a non-parametric threshold of *z* = 3.1 and a corrected cluster significance of *p* = 0.01.

## 4 | RESULTS

### 4.1 | Cortisol concentrations

Average pre-scan cortisol concentrations were 0.1593 nmol/mL (standard deviation = 0.1257), and average post-scan cortisol concentrations were 0.1603 nmol/mL (standard deviation = 0.1546). There were no significant differences between pre-scan and post-scan cortisol concentrations (*t* = −0.027, *p* = 0.97). Time of day of the scan was not associated with baseline cortisol concentrations (*R* = 0.23, *p* = 0.27) or change in cortisol (*R* = −0.013, *p* = 0.95). Cortisol concentrations were not found to be associated with smoking severity or craving (see Supplemental Materials).

### 4.2 | Cue reactivity neuroimaging

**FIGURE 1:**
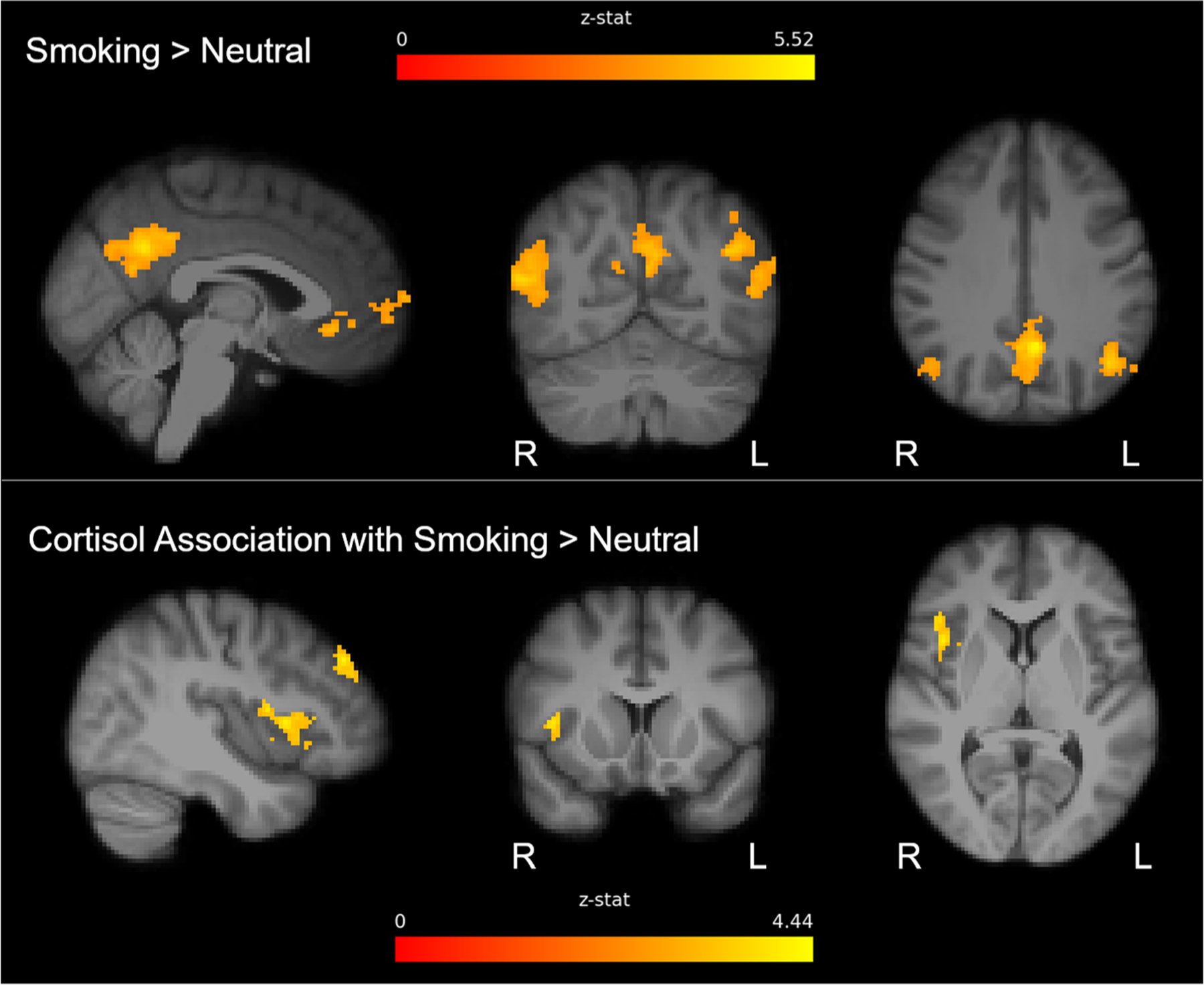
Clusters of activation for the smoking cue > neutral contrast (top row) and clusters where BOLD response for the smoking cue > neutral cue contrast is correlated with a change in pre- to post-scan cortisol (bottom row). Voxelwise Z-stat values generated by FSL’s “FLAME1” (*z* = 3.1, *p* = 0.01) overlay significant clusters. L, left; R, right.

Increased BOLD activation in the smoking > neutral cues contrast was observed in the posterior cingulate cortex, right middle temporal gyrus, left angular gyrus, left superior temporal gyrus and a cluster in the medial frontal gyrus including sections of medial prefrontal cortex and subgenual anterior cingulate cortex [Figure 1 (top), cluster information in Table 2]. The results also showed a cue-induced rise in cortisol associated with increased BOLD activation to the smoking > neutral cues contrast. Clusters associated with cue-induced cortisol included sections of the right anterior insula extending into the right frontal operculum, as well as two separate clusters within the right dorsolateral prefrontal cortex [Figure 1 (bottom); cluster information in Table 3].

## 5 | DISCUSSION

### 5.1 | Overview

Across all participants, fMRI revealed a pattern of brain reactivity to smoking versus neutral cues in medial prefrontal, posterior cingulate and bilateral parietal cortex—regions associated with the default mode network (DMN^32,33^). This finding is notable for several reasons. First, there are now numerous studies showing smoking cue-induced activation of DMN-related brain regions, demonstrating that this activation pattern is reproducible across sites.^5,7,10,29^ Second, given that the DMN is strongly implicated in internally directed cognition such as episodic memory, evoked emotion and introspection,^34^ it is plausible that smoking cues provoke such underlying cognitive processes, which then contribute to the motivation to smoke. Further evidence supporting this notion is that enhanced DMN function has been linked with greater craving and cognitive impairment during nicotine withdrawal.^35^ This 2012 study, along with the present findings, suggest that both nicotine withdrawal and smoking cues evoke a DMN-dominant state that may create barriers to smoking cessation.

Although the DMN-related activity is notable for the reasons stated, the focus of this study was to define relationships between smoking cue-induced brain reactivity and the cortisol response. These data show that the cortisol response was associated with cue-induced increases in the right insula and right DLPFC. Many studies have demonstrated that the insula is involved in both smoking relapse^4,5,36,37^ and craving.^38,39^ The critical role of the insula in maintaining drug-seeking behaviours is most clearly demonstrated by a clinical study in which patients who suffered a stroke in the insula were able to more easily quit and reported little or no urge to smoke.^40^ These findings are supported by rodent work where pharmacological inactivation of the insula attenuates nicotine-seeking behaviour in response to cues and nicotine-priming injections.^41^ Lesion-associated increases in functional connectivity of the dorsal cingulate, lateral prefrontal cortex and insula and decreases in the medial prefrontal cortex and temporal cortex have also been tied to successful smoking remission,^42^ suggesting that regions such as the insula may be part of a larger network playing a role in the maintenance of substance use. The insula is not only involved in mediating nicotine dependence but also plays a role in interoception^43^ and is a well-established core region of the salience network that is known to integrate internal and external information to drive behavioural responses.^44^ Because of the insula’s demonstrated role in interoception and salience, this region activates across several tasks involving emotion, sensation and executive function^43,45,46^ and implies that the insula could be involved in multiple cognitive aspects relevant to cue reactivity and motivating subsequent behaviours. For example, the insula may redirect attention toward drug-taking after viewing cues,^36^ be involved in the recall of memory for smoking cues^28^ or may be associated with feelings of craving induced by cues.^47,48^ Cue reactivity in the insula has also been tied to genetic factors involved in reward processing.^49–51^

Cortisol may also play a significant role in salience and interoception. Schulz et al^52^ proposed that acute stress activates the hypothalamic–pituitary axis to release cortisol, which then leads to enhanced interoception. The cortisol response may enhance vigilance and detection of salient stimuli in the environment (including internal urges) by heightening the sensitivity of the amygdala.^53^ Furthermore, the priming effect of the cortisol response on craving urges is complex. Although the cortisol response after a social stress task is followed by greater urges to smoke, a relatively blunted cortisol response (less strong cortisol responses) seems to be associated with the greater urges.^54^ Similarly, other types of interoceptive tasks, such as heartbeat tracking, have shown that the stress-induced cortisol response is linked with better accuracy,^55^ yet people who smoke perform worse on this task than controls.^56^ Since the cortisol response has been linked with the perception of internal and external cues and is responsive to a variety of environmental conditions, it is important to study as a biomarker in substance use populations.

In addition to the insula, the present study demonstrates a link between cue-induced cortisol and DLPFC activity, which may suggest that those with greater cue-induced cortisol responses are more engaged with viewing smoking cues, relative to neutral cues. The DLPFC is thought to integrate contextual information about the drug of interest, such as drug reward and availability, the incentive salience of drug-related cues, as well as ongoing cognitive and emotional processes that may inform decision-making.^57,58^ DLPFC reactivity to cues may also direct greater attention toward smoking cues relative to neutral cues. Collectively, the anterior insula and DLPFC are thought to coordinate task performance in addition to their potential roles in salience detection,^59,60^ as greater attentional bias toward drug cues (vs. concurrent neutral cues) is associated with greater DLPFC activation,^47^ whereas inactivation of the DLPFC via transcranial magnetic stimulation reduces cue-induced craving.^61,62^ These studies demonstrate that DLPFC function is related to cue-induced craving and that the craving may be a byproduct of enhanced attentional processes recognising the salience of smoking cues.

### 5.2 | Potential role of cortisol

Nicotine-dependent individuals appear to have elevated tonic cortisol concentrations compared to people who do not smoke.^19^ These elevated cortisol concentrations decline immediately during a quit attempt in those who return to use.^11^ Such a reduction in cortisol may be directly related to drug use, as acute administration of nicotine^63,64^ and cigarette smoking^17^ increase cortisol concentrations. Drug-paired cues also induce a cortisol response,^65^ which is consistent with a number of studies showing that neuroendocrine function can be classically conditioned in humans.^66–69^ Although we did not observe a cue-induced rise in cortisol at the group level, the individual variability in cortisol responsiveness in our sample was sufficient to yield an association with brain activity. The link between drug cues and cortisol is clinically relevant as greater amounts of cue-induced cortisol release are linked to poorer treatment outcomes.^13^ It has been demonstrated that intravenous^23^ and oral^70^ cortisol administration in the laboratory induces a craving response in cocaine- and heroin-dependent individuals, respectively, and so it is plausible that a rise in cortisol may drive craving and relapse, regardless of the source of the factor driving the rise. This link between cortisol and drug administration is further supported by rodent work where a decrease in central nervous system glucocorticoid receptors eliminates cocaine self-administration.^21^ Although many questions concerning the role of cortisol changes in relapse remain, the present study offers new insights into the complex relationship by linking a cue-induced increase in cortisol to brain activation in regions associated with cue reactivity and relapse vulnerability.

## 6 | LIMITATIONS

The present study establishes a link between the cortisol response and brain reactivity to cues; however, the causal relationship between the two is not yet established, as these findings are based on Pearson correlations. Nevertheless, the observed association bridges the gap between two literatures that strongly support the importance of both cortisol and smoking cues in addiction. The sample size within this cohort could be considered a limitation; however, the stringent thresholds used for cluster formation and significance (*z* = 3.1, *p* = 0.01) by FSL’s “FLAME1” are thought to provide conservative voxel-wise inference and lower family-wise error rate when compared to other software packages.^71^ Furthermore, these findings occurred in regions that have been closely associated with cue reactivity and relapse vulnerability in previous work. Taken together, these considerations lead us to be confident that our findings are reliable, and potentially important biomarkers in human substance use disorders. Although the balance of sex within our study was skewed toward men, we did not observe any effects of sex and would likely be under-powered to observe potential effects.

## 7 | CONCLUSIONS

The results of the present study establish a link between increased cortisol and smoking cue reactivity in the insula and DLPFC of tobacco-smoking individuals. This relationship provides support for the idea that individuals who mobilise metabolic resources via cortisol in response to drug cues also have greater activity in brain regions that subserve interoceptive awareness, salience and attentional processing of smoking cues. These findings suggest a mechanism mediating individual responses to smoking cues and thus identify plausible targets for interventions that may alleviate drug-seeking or prevent relapse in individuals seeking to quit smoking.

## Supplementary Material

Supplementary Material

## Figures and Tables

**TABLE 1:** Demographic information for participants.

Demographic variable	
Female	9
Male	18
Age	27.0 ± 5.9
FTND	4.6 ± 2.2
Years of smoking	10.2 ± 6.7
Cigarettes per day	11.4 ± 6.1
Ethnicity	
Asian	5
Asian & White	1
Black/African American	1
More than one race	1
White/Caucasian	19
Hispanic	3
Non-Hispanic	24

Abbreviations: (±), standard deviation; FTND, Fagerström Test for Nicotine Dependence.

**TABLE 2:** Clusters of activation for the smoking cue > neutral cue contrast.

Brain region	# Voxels	*x* (COG)	*y* (COG)	*z* (COG)	*p*-value	Brodmann areas	Anatomical structure
Posterior cingulate cortex	888	−1.98	−57.4	29.7	<0.0001	31	Cingulate gyrus
R. lateral parietal	707	53.7	−66.2	18.5	<0.0001	19	Middle temporal gyrus
L. lateral parietal	433	−45.6	−66.1	36.5	<0.0001	39	Angular gyrus
L. lateral parietal	289	−54.4	−64.3	13.5	<0.0001	22,39	Middle/superior temporal gyrus*
Medial prefrontal cortex	276	−0.862	51.3	−2.15	0.0001	10,32	Medial frontal gyrus*

**TABLE 3:** Clusters where BOLD response for the smoking cue > neutral cue contrast is correlated with a change in pre- to post-scan cortisol.

Brain region	No. of voxels	*x* (COG)	*y* (COG)	*z* (COG)	*p*-value	Brodmann areas	Anatomical structure
*R. Insula*	234	41.2	12.7	4.63	0.0002	13	Insula
R. DLPFC	183	37.5	40.2	34.2	0.0013	9	Middle frontal gyrus
R. DLPFC	136	24.8	59.8	21.8	0.0081	9	Superior frontal gyrus

*Note*: Coordinates are listed for MNI space.

Abbreviations: COG, center of gravity; L, left; R, right.

## Data Availability

The data that support the findings of this study are available on request from the corresponding author. The data are not publicly available due to privacy or ethical restrictions.
