# Supplementary material for: Brain and cortisol responses to smoking cues are linked in tobacco-smoking individuals
Source: Addict Biol. Author manuscript; Available in PMC 2024 Dec 1. (PMC11572701; doi:10.1111/adb.13338)
Supplement: Supplementary Material [file NIHMS2028820-supplement-Supplementary_Material.docx]

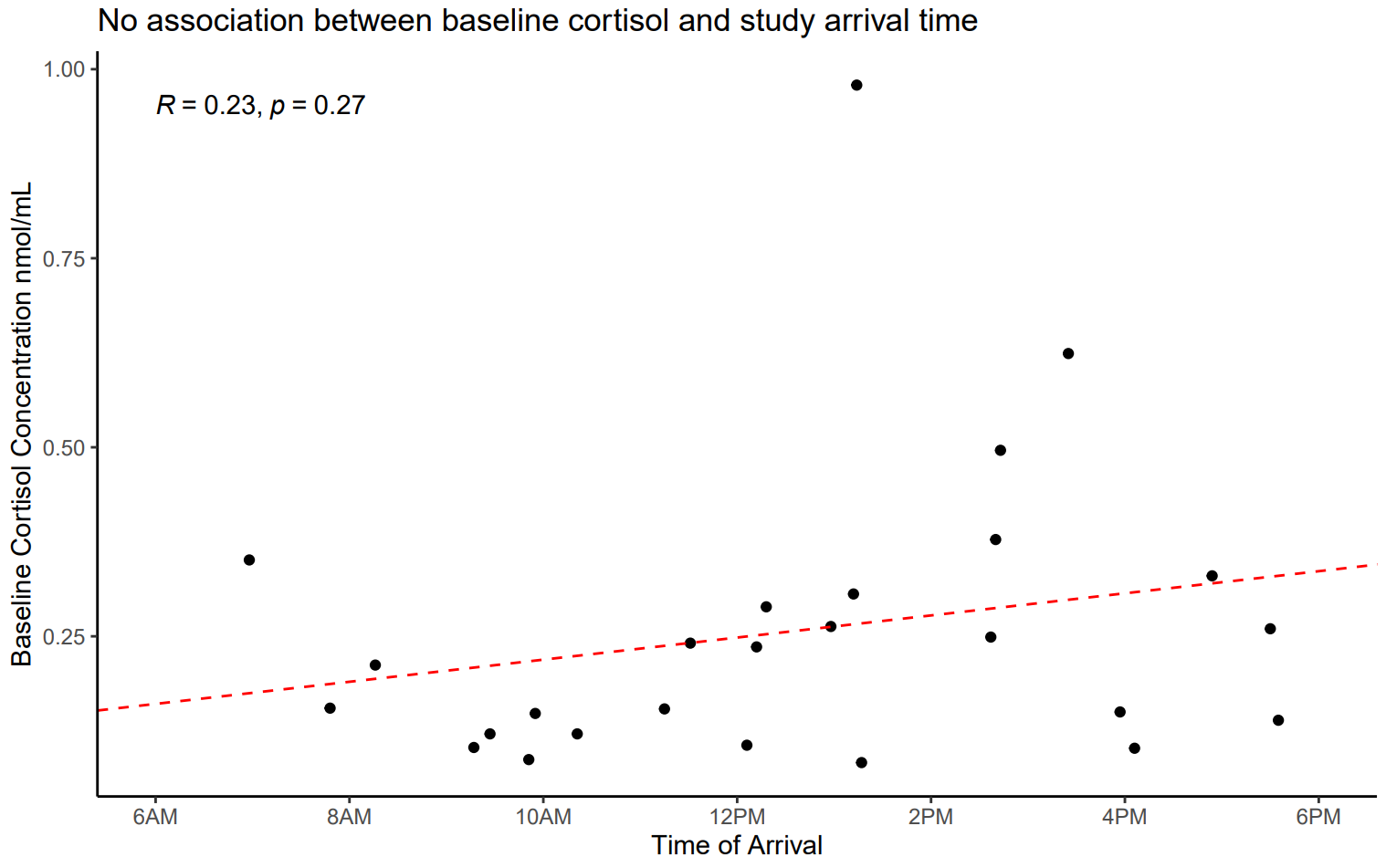


Figure S1. Scatterplot of baseline cortisol concentrations and time of arrival. Linear regression reveals no significance (R = 0.23, p =0.27).


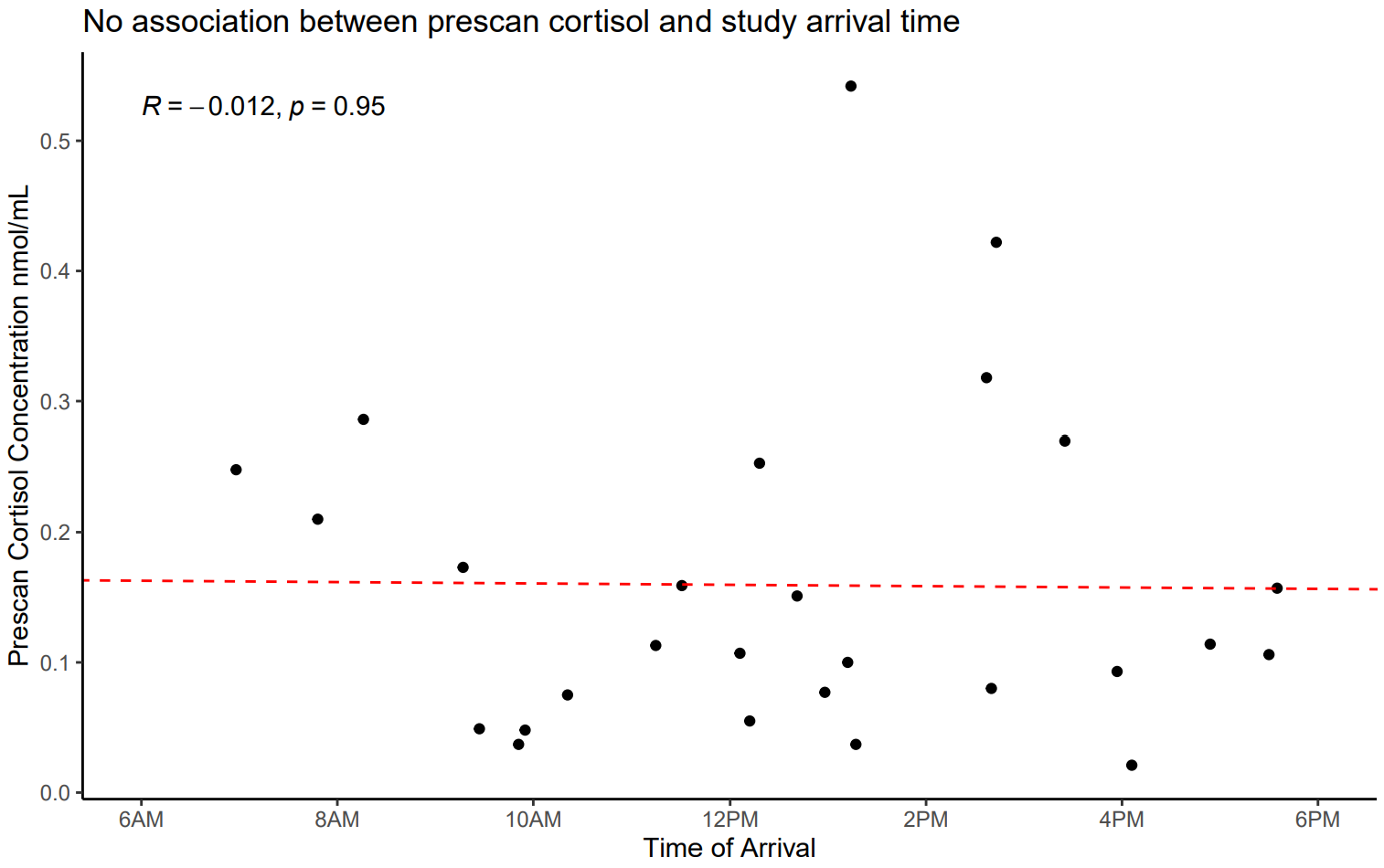


Figure S2. Scatterplot of prescan cortisol concentrations and time of arrival. Linear regression reveals no significance (R = -0.012, p =0.95).


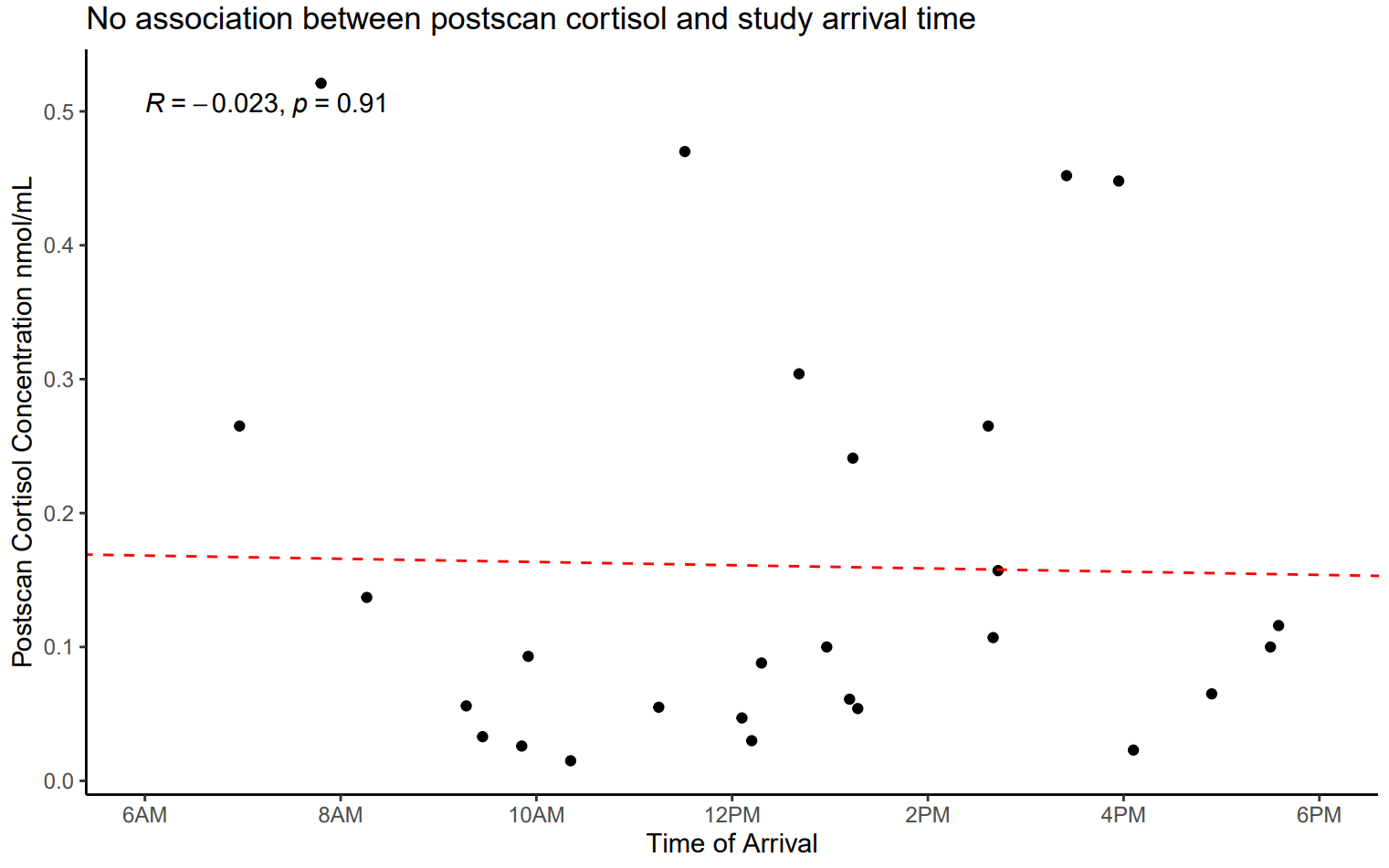


Figure S3. Scatterplot of postscan cortisol concentrations and time of arrival. Linear regression reveals no significance (R = -0.023, p =0.91).


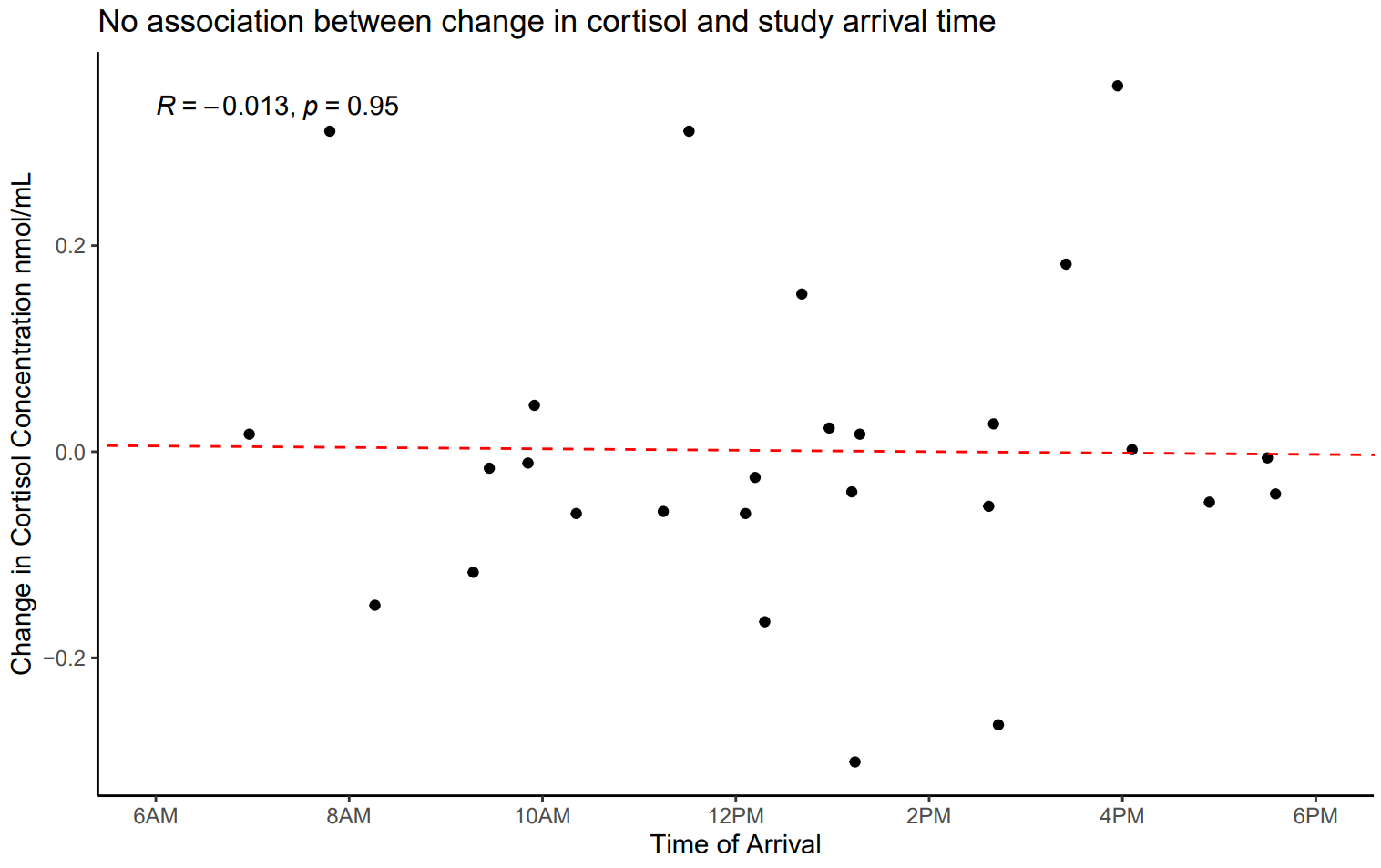


Figure S4. Scatterplot of change in cortisol concentrations (postscan – prescan) and time of arrival. Linear regression reveals no significance (R= -0.013, p =0.95).


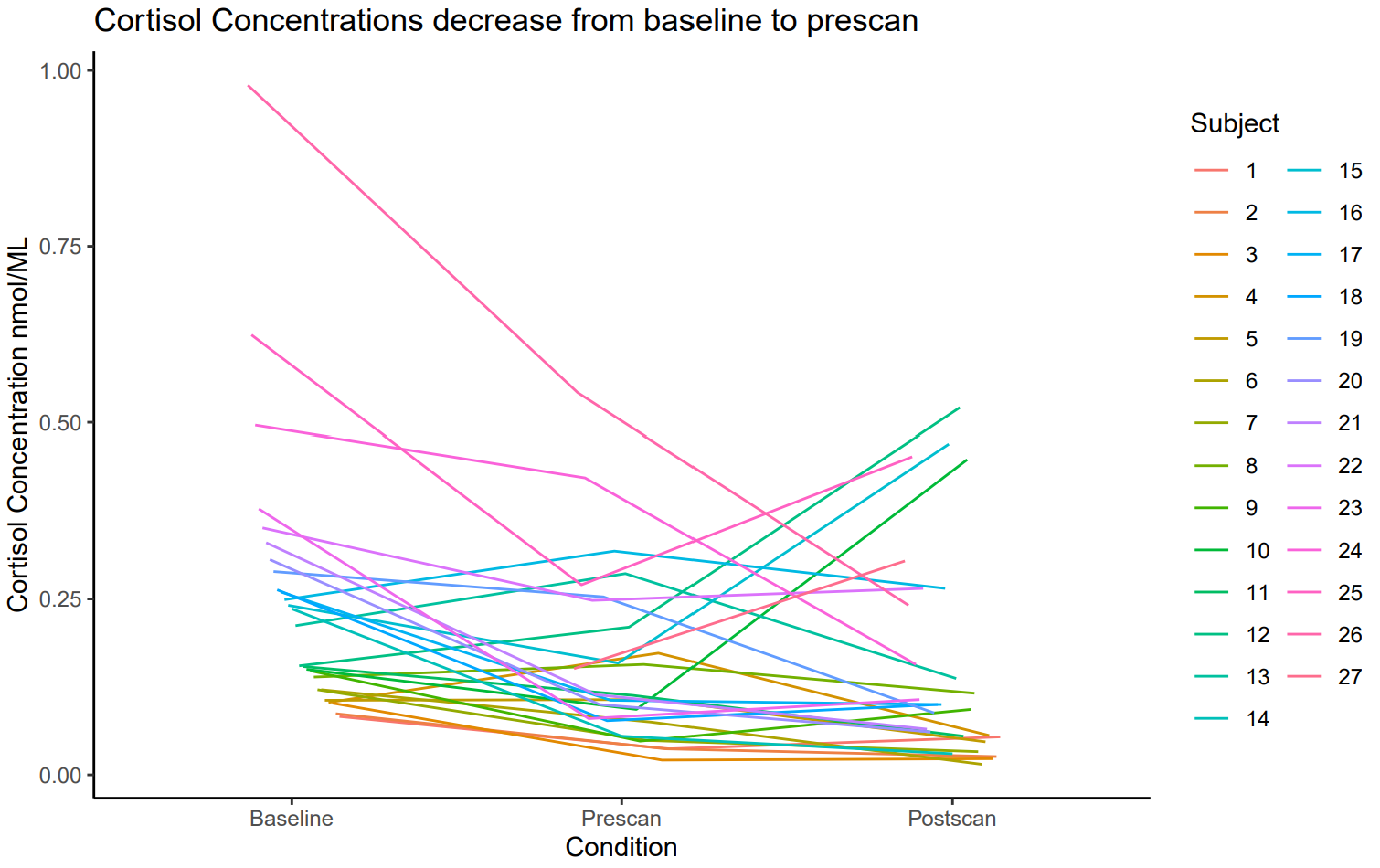


Figure S5. Cortisol concentrations at baseline, prescan, and postscan for each subject. Each subject’s data is offset at the x-axis nodes to enhance visibility.

An ANOVA of cortisol concentration at the three timepoints was barely significant (F = 3.185, p = 0.0469). Posthoc Tukey HSD tests revealed a trend toward significance where baseline cortisol had greater concentrations than prescan (p = 0.077) and postscan (p = 0.081) measurements, but no significance was observed between postscan and prescan averages (p = 0.97).


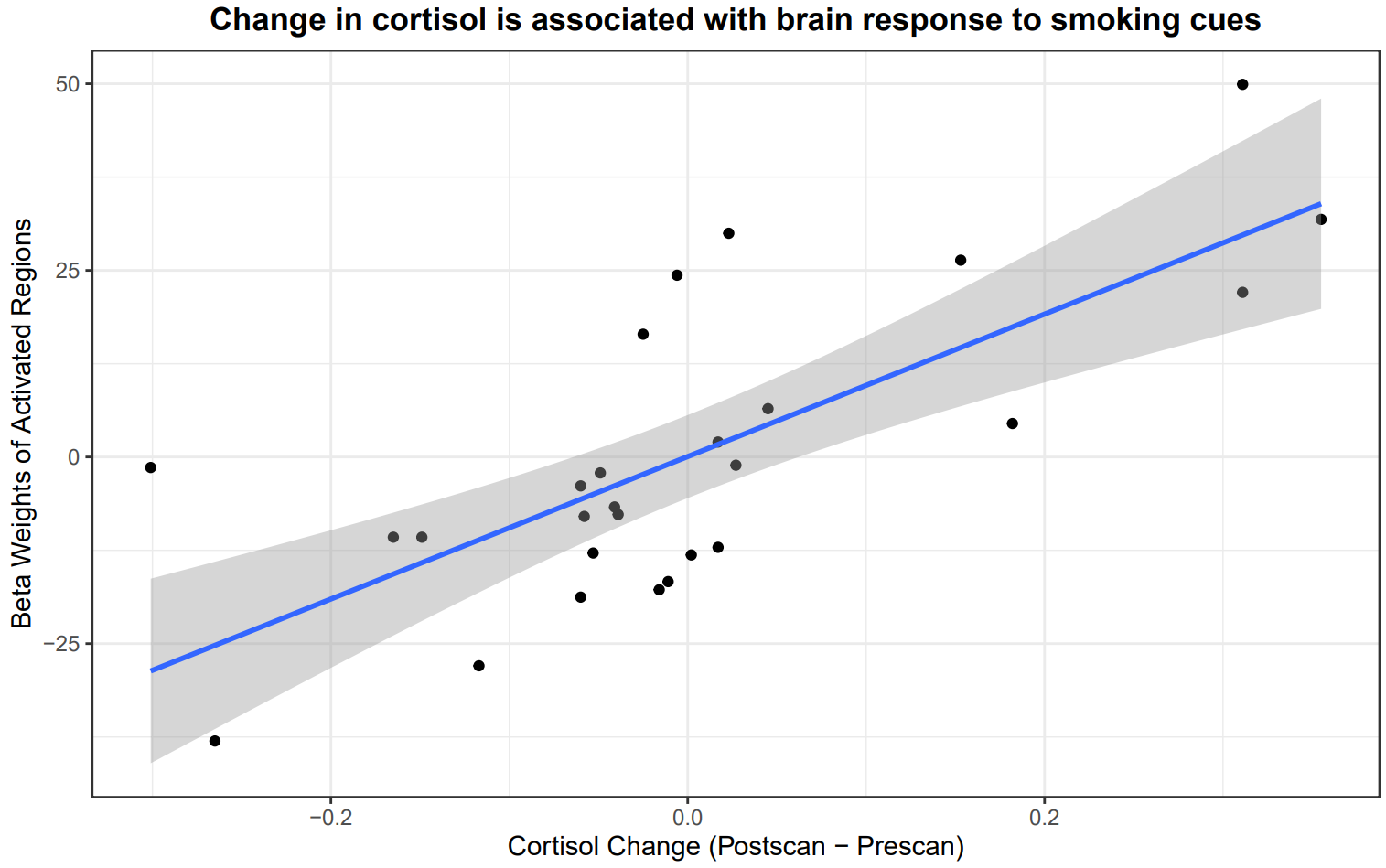


Figure S6. Change in cortisol (postscan – prescan) is associated with activated brain regions during a cue reactivity task (smoking cues > neutral cues). Gray boundaries represent 95% confidence intervals.


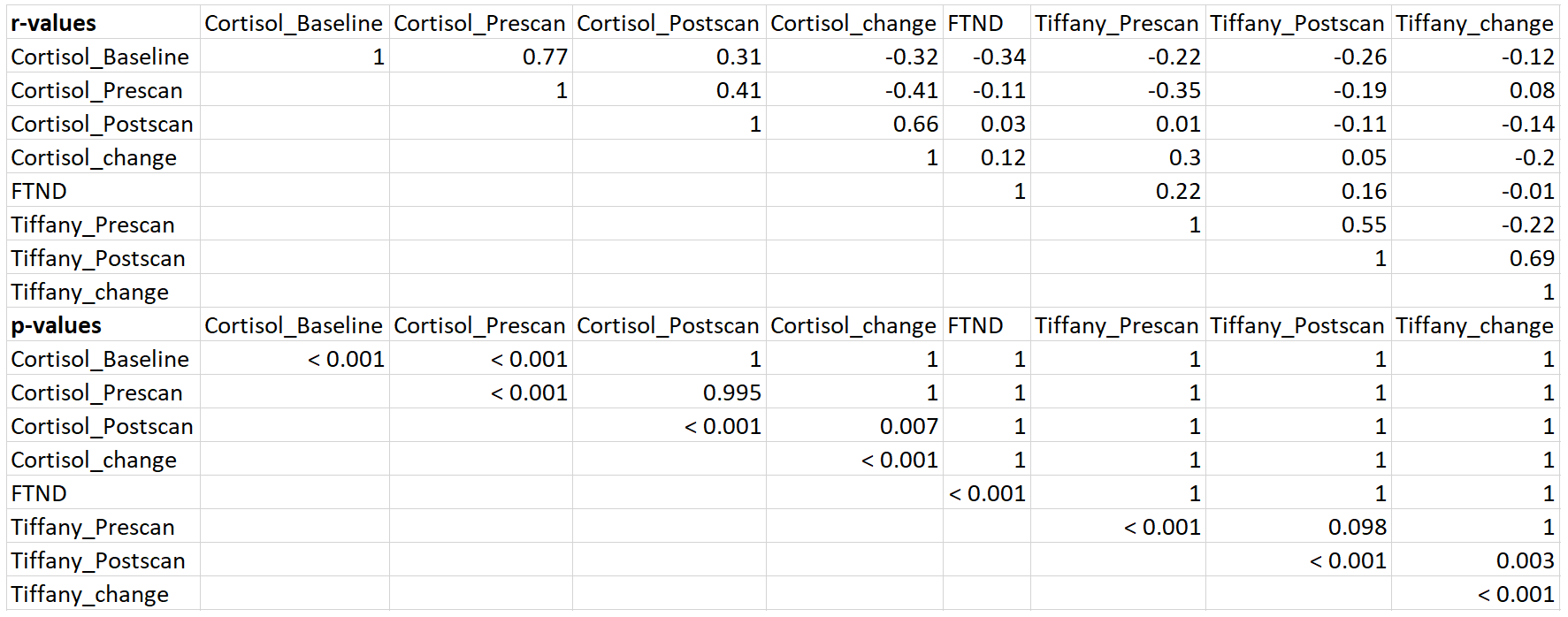


Table S1. Correlation matrix including cortisol measurements (baseline, prescan, postscan, change), Fagerstrom Test for Nicotine Dependence (FTND), and Tiffany scores (prescan, postscan, change). Pearson’s correlation r values are listed in the top half, while Bonferroni-corrected p-values are listed below.
